# Supplementary material for: Exploring functional protein covariation across single cells using nPOP
Source: Genome Biol. 2022 Dec 16;23:261. doi: 10.1186/s13059-022-02817-5 (PMC9756690; doi:10.1186/s13059-022-02817-5)
Supplement: Supplementary file 1 — Additional file 1: Supplemental figures S1-S16. [file 13059_2022_2817_MOESM1_ESM.pdf]

Additional file 1: Supplementary figures

Exploring functional protein covariation across single cells using nPOP

Andrew Leduc,[1] R. Gray Huffman,[1] Joshua Cantlon,[2] Saad Khan,[1] & Nikolai Slavov[1,✉]

[1]Departments of Bioengineering, Biology, Chemistry and Chemical Biology, Single Cell Proteomics Center, and Barnett Institute, Northeastern University, Boston, MA 02115, USA
[2]Scienion AG, Phoenix, AZ 85042, USA
✉ Correspondence: nslavov@alum.mit.edu or nslavov@northeastern.edu
∈ Data, code & protocols: scp.slavovlab.net/nPOP

This file contains all supplementary figures (Fig. S1-S16) with corresponding figure legends. Additional information can be found at scp.slavovlab.net/nPOP.

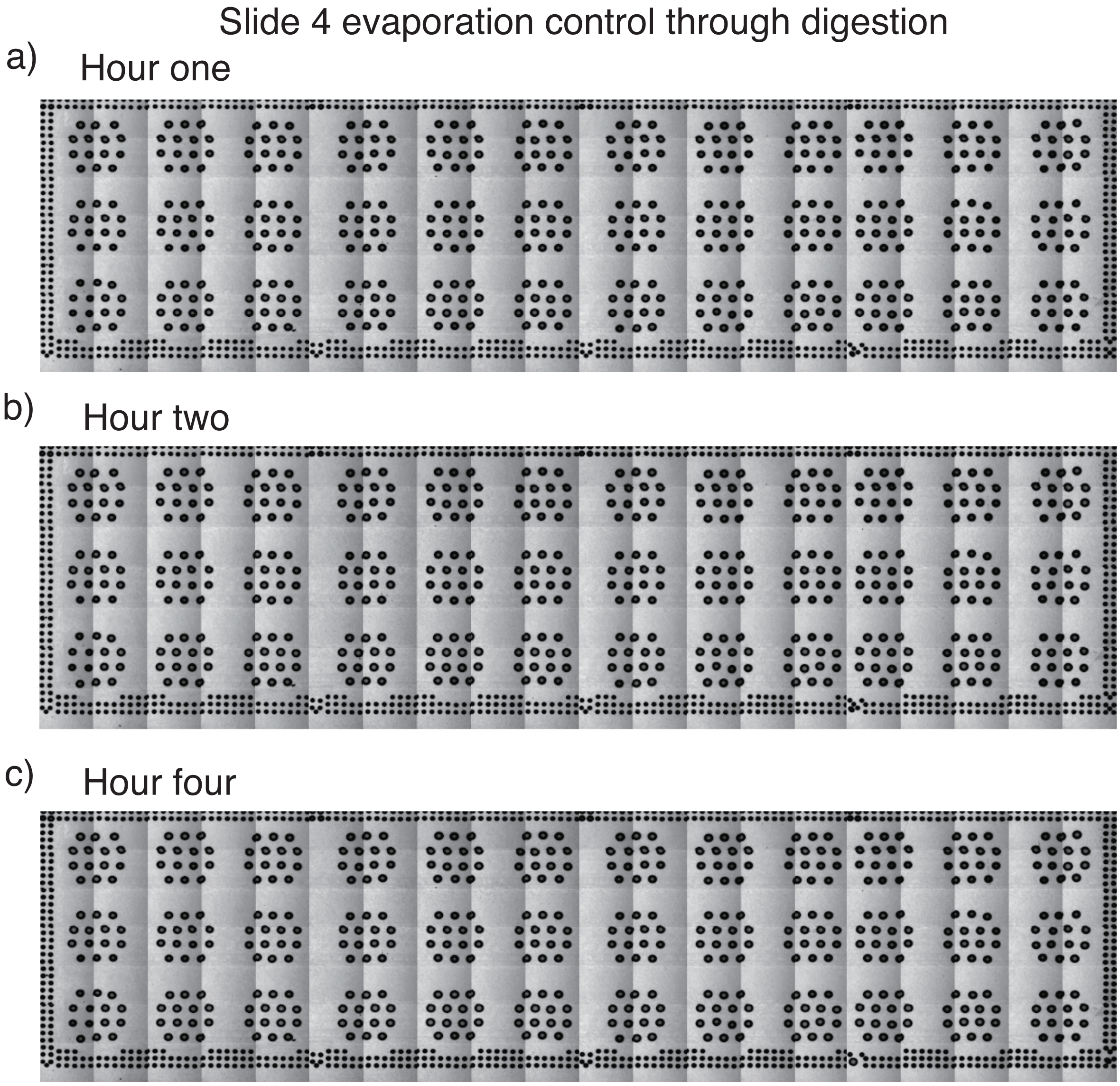

Figure S1 | Evaluating droplet evaporation. Droplets on glass slide after adding DMSO, single cells, and digest mix after (**a**) one hour, (**b**) two hours, and (**c**) four hours.

Sample preparation	mPOP	nanoPOTS	N2	proteoCHIP	nPOP
Volume (nl)	1000-1500	100-200	30	100-150	20-30
Wells/ drops per cluster	N/A	N/A	11 or 16	16	Any
Cells per single prep (Demonstrated)	324	48	108	170	1,556
Multiplexing, cell per one MS run (Demonstrated)	11, 16	LF, 11	11, 16	16	3, 16, 18
Sample cleanup (Demonstrated)	None	Trapping column	Trapping column	FAIMS	None
Autosampler compatibility	384 well plate	Custom	Custom	Custom	384 well plate

Figure S2 | Table listing various important aspects of sample preparation for different single cell sample preparation methodologies.

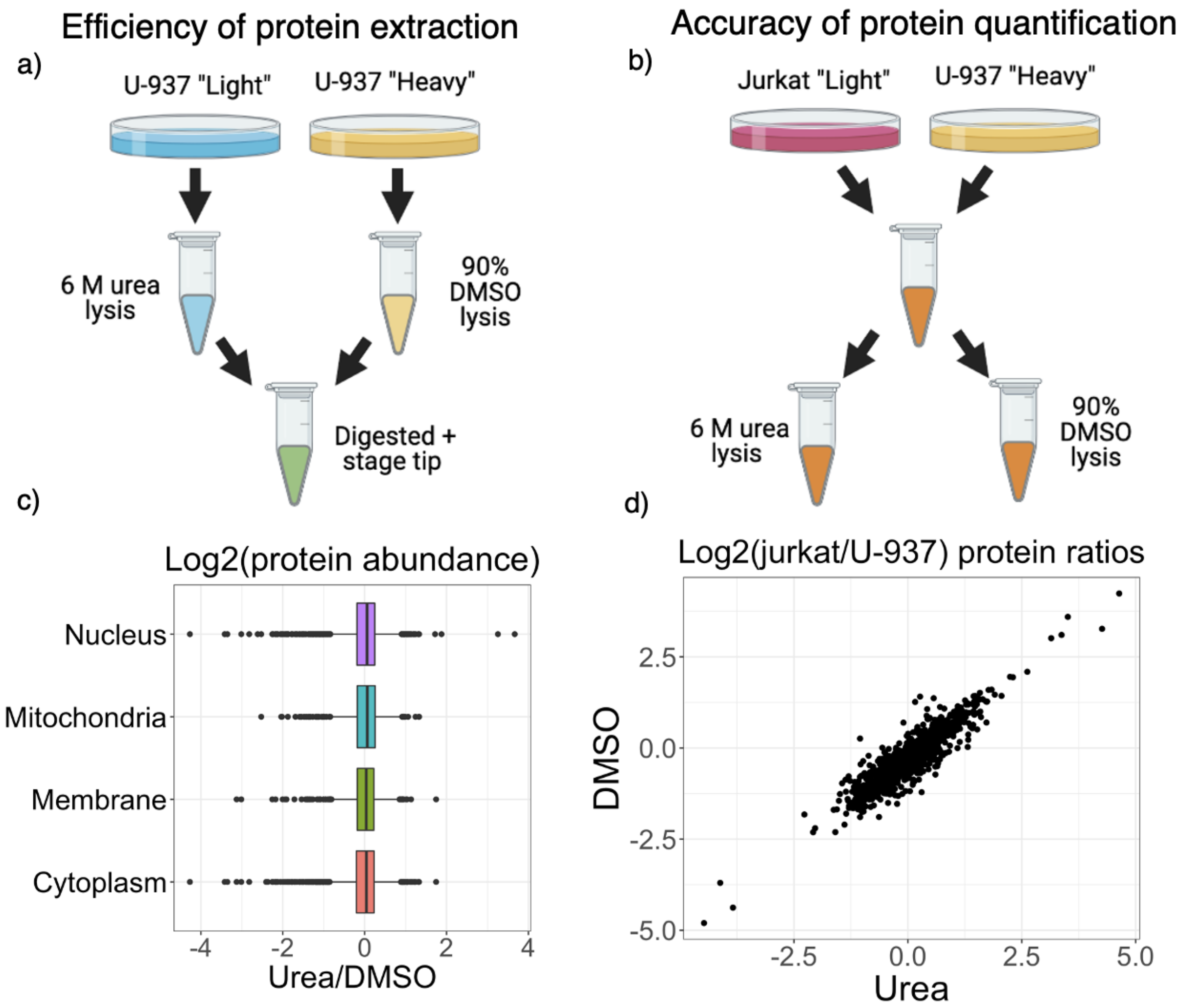

Figure S3 | Evaluating the efficiency of protein extraction by DMSO cell lysis. (**a**) Equal number of U-937 cells labeled with "Light" and "Heavy" isotopes via SILAC were lysed with urea or DMSO, diluted, and combined for digestion. The SILAC ratios for proteins from different cellular compartments show comparable protein recovery for DMSO and urea cell lysis. (**b**) Equal number of SILAC labeled "Light" Jurkat and "Heavy" U-937 cells were combined, and the mixed sample was then divided for cell lysis either by urea or or by DMSO. The agreement between the SILAC ratios from the two methods supports the use of DMSO lysis for quantitative protein analysis.

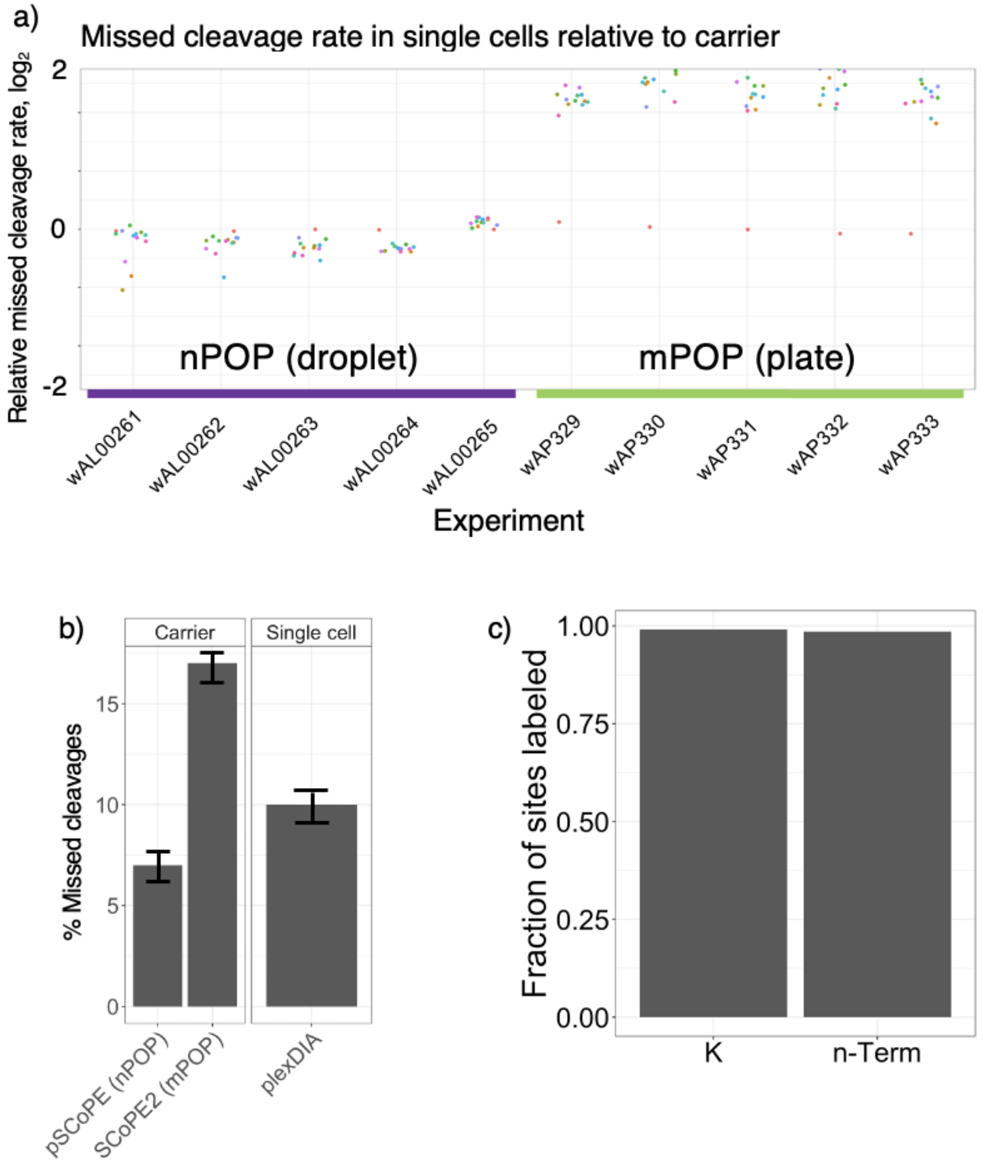

Figure S4 | Single cell digestion evaluation. (**a**) The ratio of intensities for peptides with missed cleavages to their corresponding fully cleaved products is calculated in both the single cells and the carrier. The ratio of these two numbers is plotted as a way to evaluate miss cleavage rate in single cells compared to the carrier. Ratios centered around 0 indicate the proteins from single cells are digested as well as the proteins from the carrier sample, which has about 3 % peptides with missed cleavages. This panels is generated by DO-MS[1] and can be found in the associated DO-MS report. Evaluating the metric in (**a**) depends on the digestion efficiency of the carrier. SCoPE2 data is taken from the data in the SCoPE2 protocol[2]. (**b**) shows that the carriers for the nPOP sets have a lower missed cleavage rate than the carrier for the mPOP sets increasing the significance of the better digestion in the single cells relative to the carrier. (**b**) also shows low missed cleavage rate for the single cells run without a carrier via DIA of $\sim 10\%$. (**c**) Fraction of labeled lysine or n-Termini sites out for many pooled single cells prepared by nPOP, labeled with TMTpro and searched with TMTpro as a variable modification.

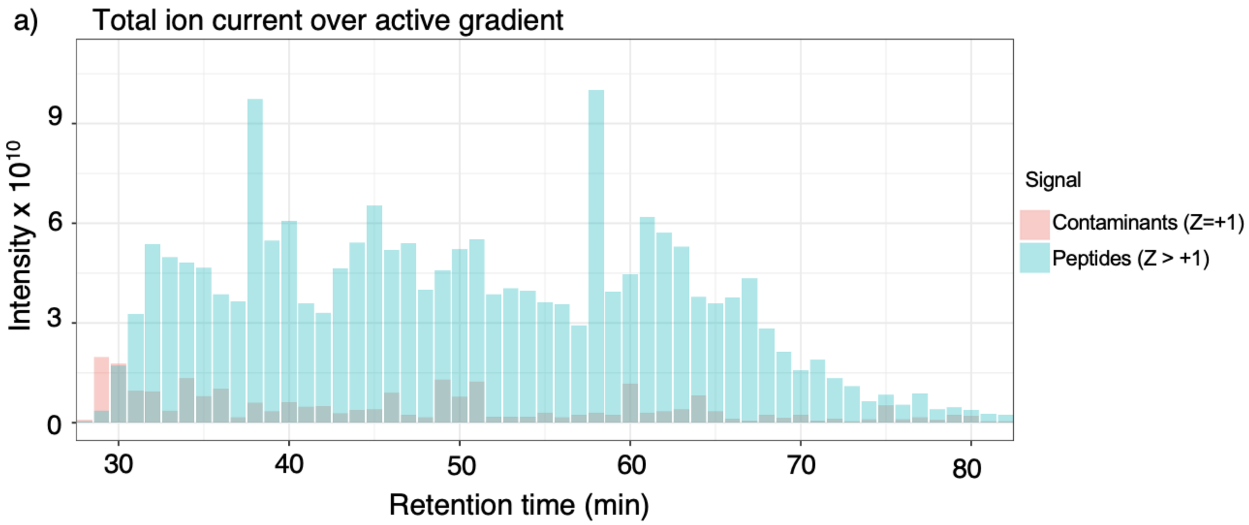

Figure S5 | Single-cell samples processed by nPOP have low contamination and complete protein digestion. (**a**) Ion current from +1 charged ions (likely corresponding to contaminants) and $\geq +2$ charged ions, likely correspond to peptides. Current from $\geq +2$ charged ions far exceeds that of +1 indicating that contamination of samples prepared by nPOP is low.

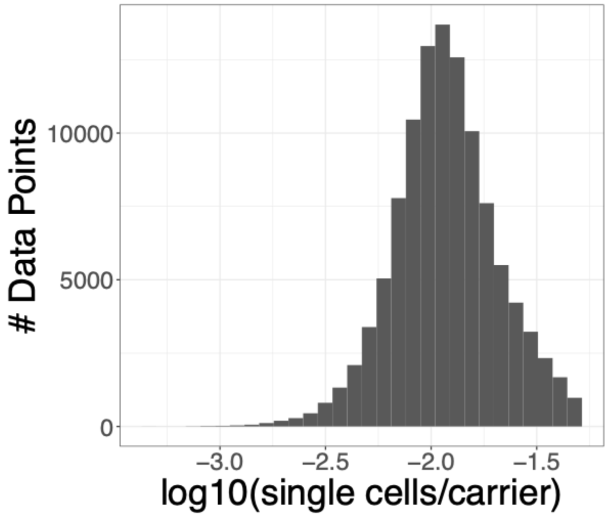

Figure S6 | Ratio of single cells (averaged abundance within a TMT set) to the carrier sample.

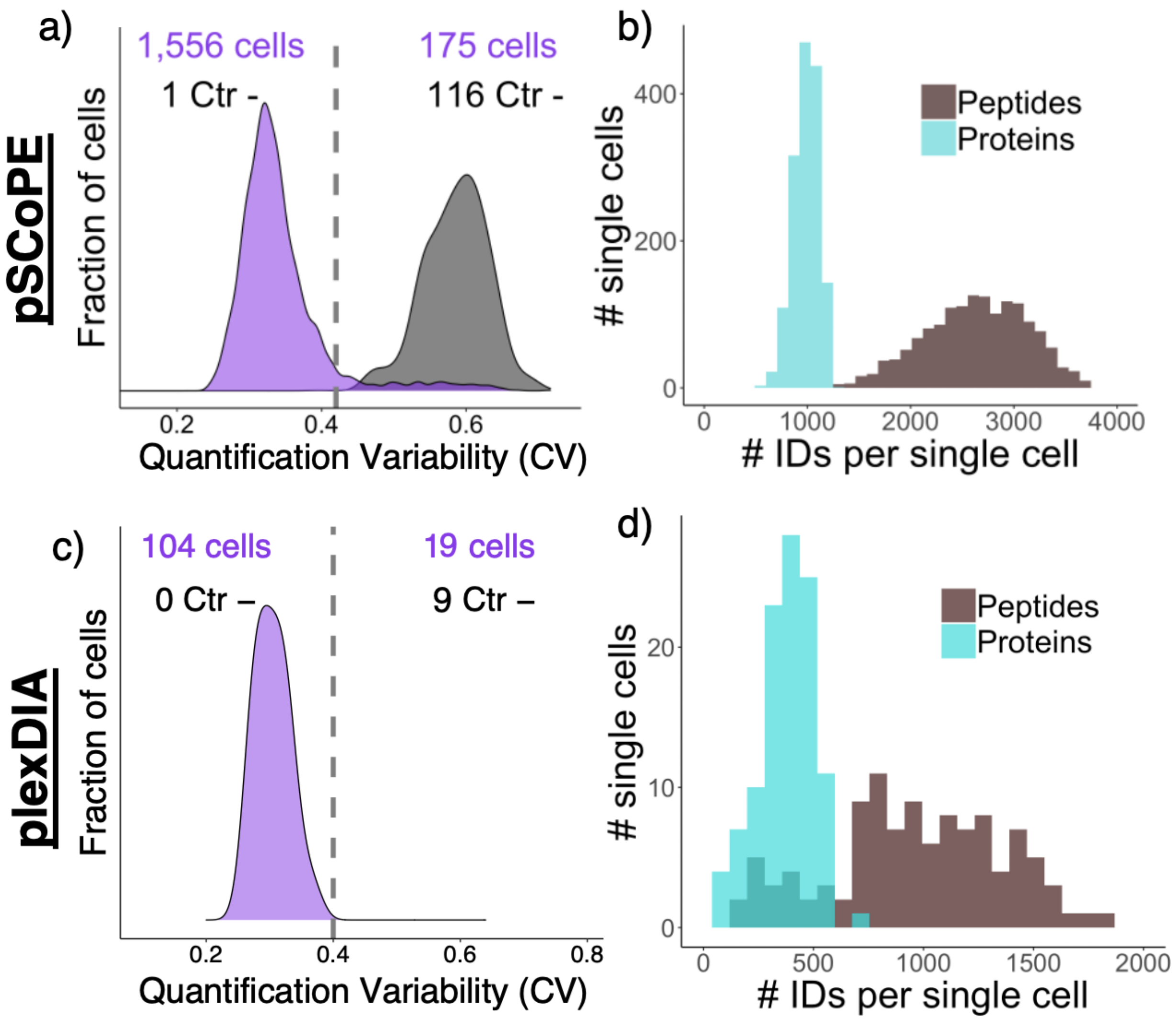

Figure S7 | Single-cell data quality controls. (**a**) Quantitative variability for single cells analyzed by pSCoPE defined as the average CV per single cell between peptides mapping to the same protein. (**b**) Number of proteins and peptides per single cells analysed by pSCoPE. (**c**) Quantitative variability, CVs as described for (**a**), for single cells analyzed by plexDIA. (**d**) Number of proteins and peptides per single cells analysed by plexDIA.

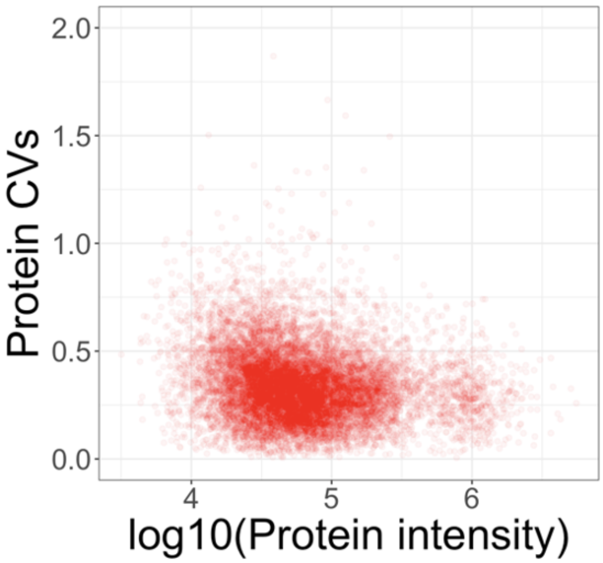

Figure S8 | CV of peptides mapping to the same protein vs intensity of the protein defined by average intensities of the peptides mapping to the protein.

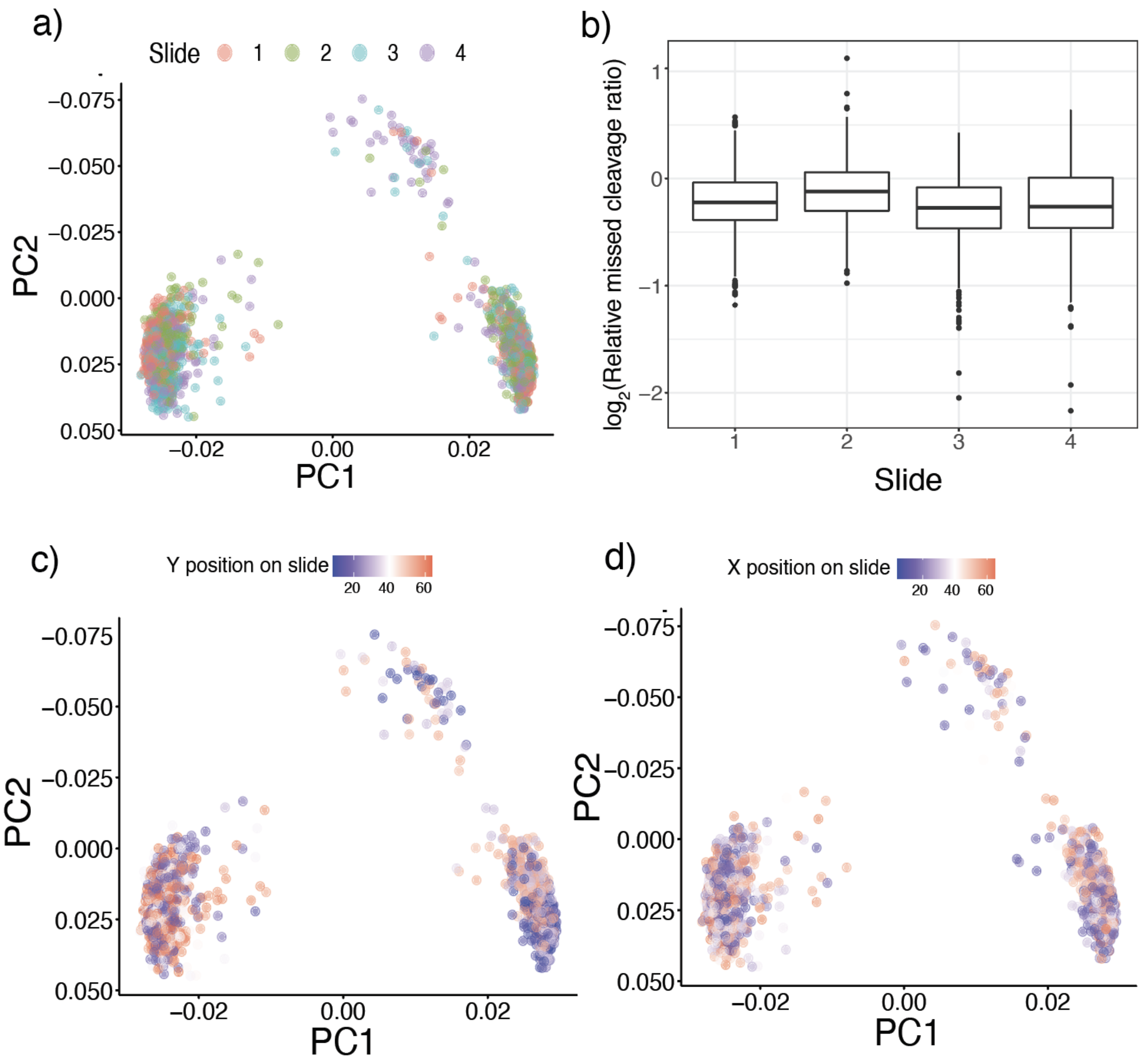

Figure S9 | Examining potential batch effects. (**a**) PCA with single cells colored by which slide they were prepared on. (**b**) Distribution of ratios for missed cleaved peptides to their cleaved counterparts in single cells compared to the carrier sample plotted by slide. (**c**) Cells colored by X coordinates (left right) within slide. (**d**) cells colored by Y coordinates (front back) within slide.

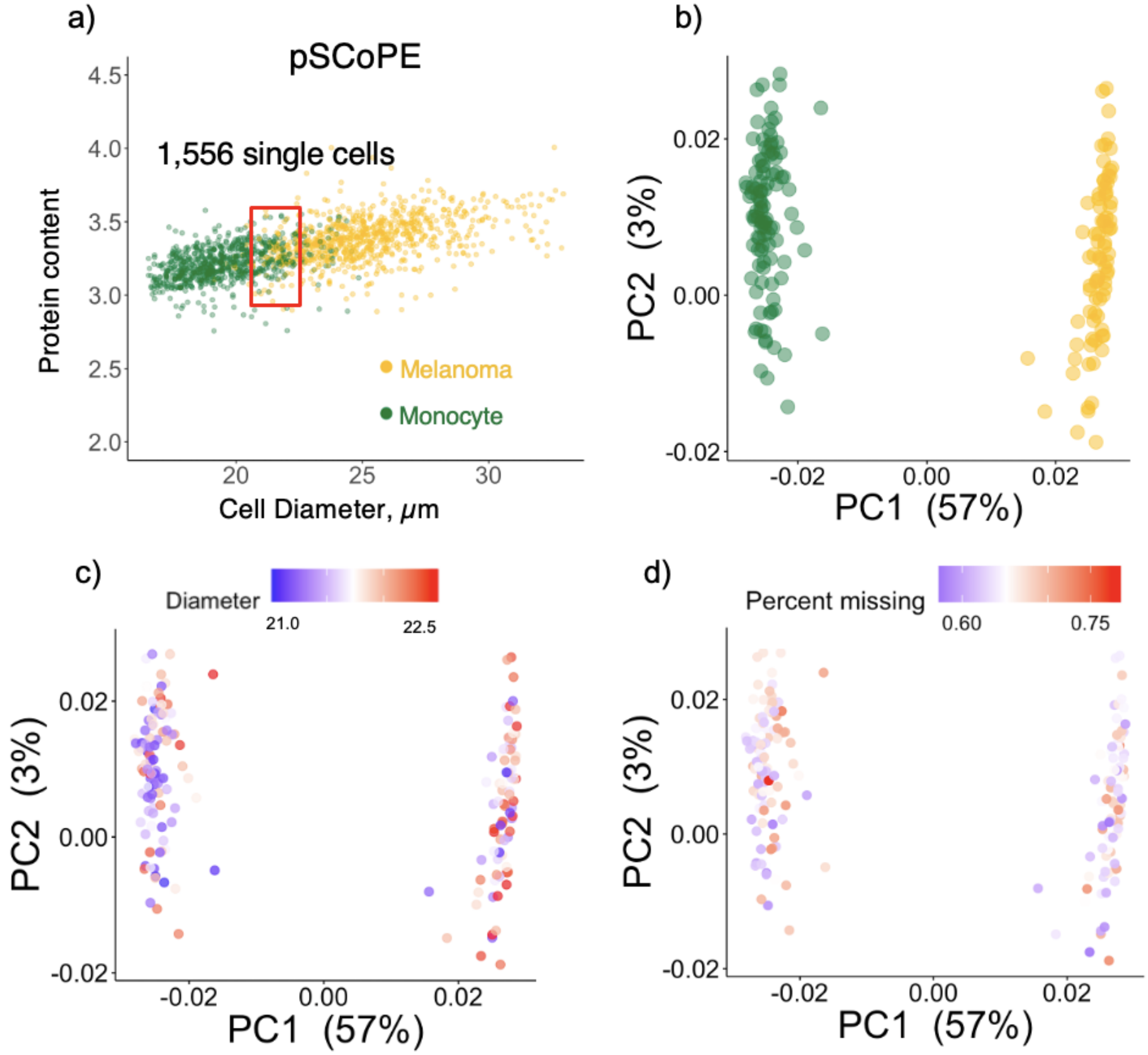

Figure S10 | (**a**) By taking a subset of Monocyte and Melanoma cells with overlapping diameter and protein content, (**b**) we can demonstrate that cell type separation is not driven by cell size. Color coding by cell size, (**c**), and missing data percentage, (**d**), shows that cell type and not size missing data is driving separation across PC1.

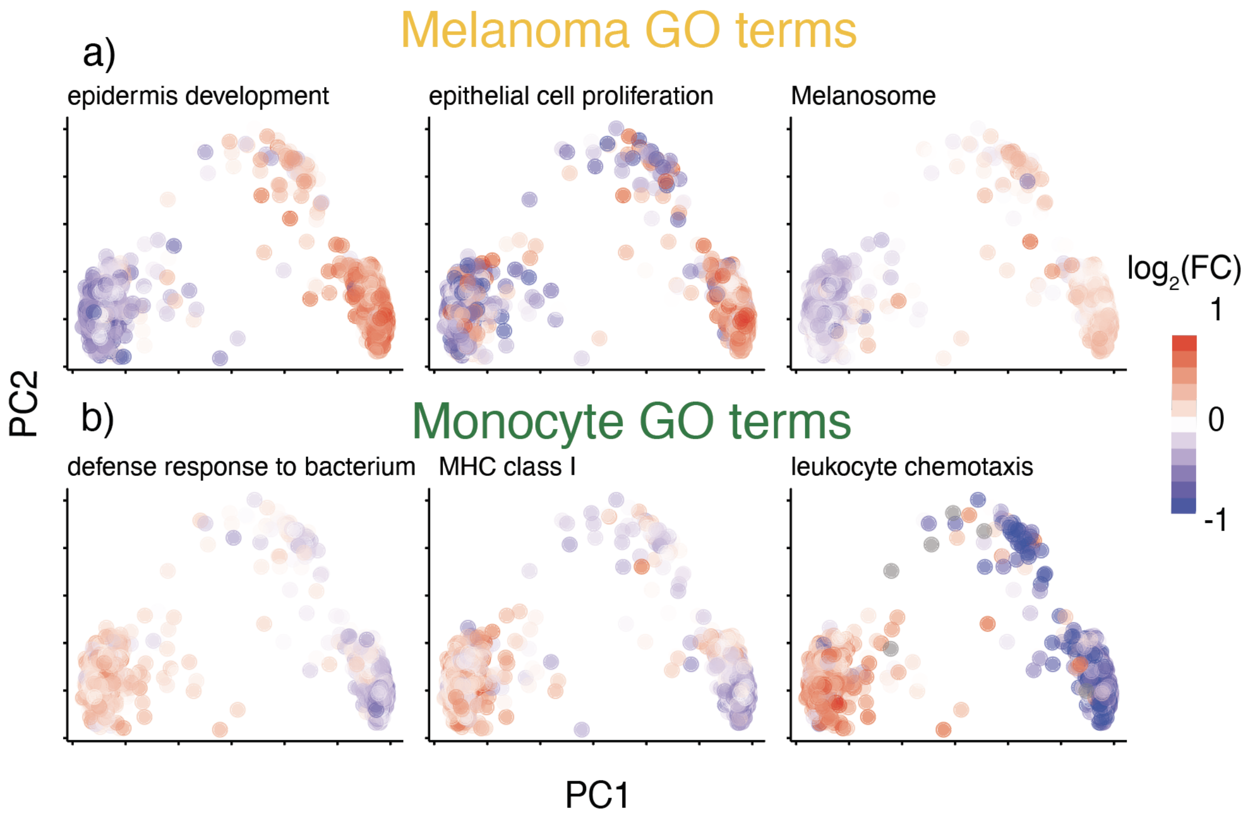

Figure S11 | **Protein set enrichment, Monocyte vs. Melanoma.** Cells colored by the median abundance of proteins from protein sets associated with melanoma (**a**) and monocytes (**b**).

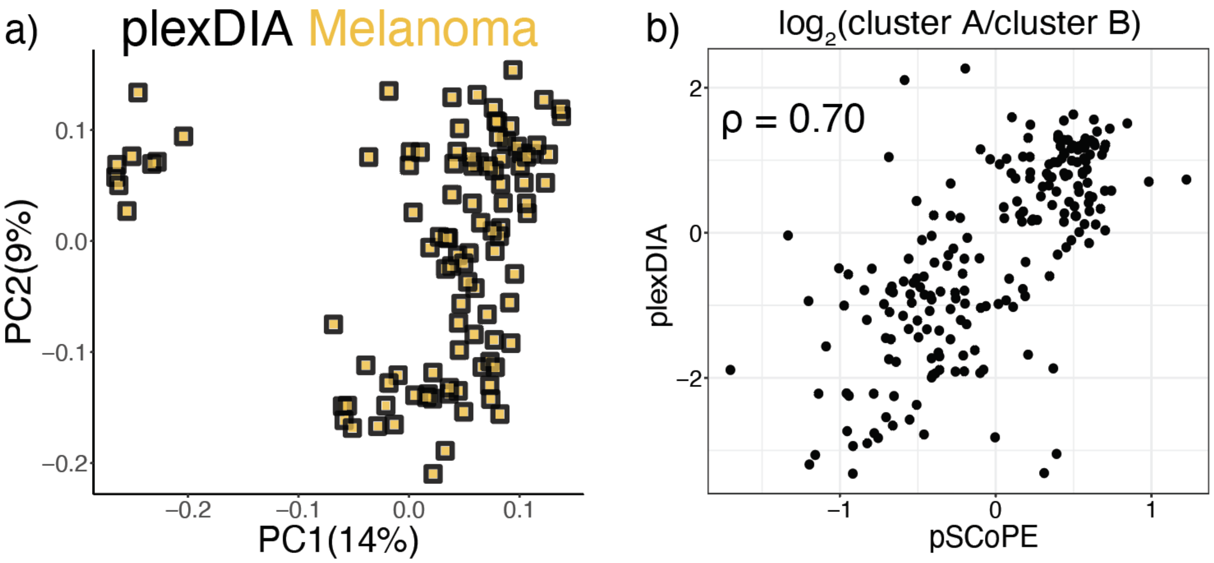

Figure S12 | Validation of two clusters. (**a**) Principal component analysis of plexDIA melanoma cells showing two distinct clusters. (**b**) Protein fold changes between each cluster plotted in plexDIA vs pSCoPE data sets show agreement.

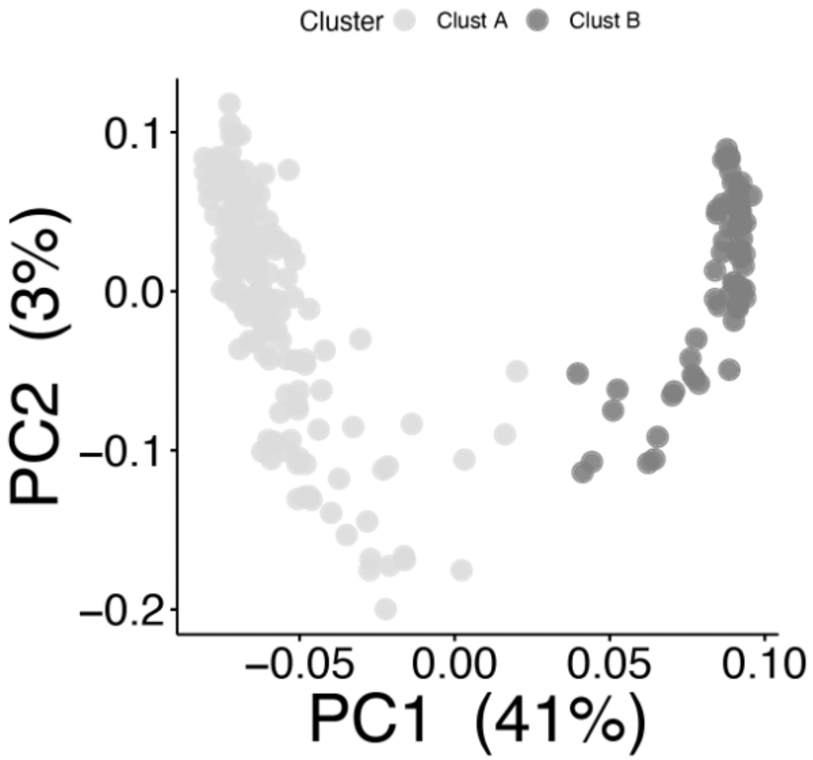

Figure S13 | Down sampling from cluster A by randomly selecting a subset of 150 cells increases fraction of variation explained by PC1, which separates the two populations.

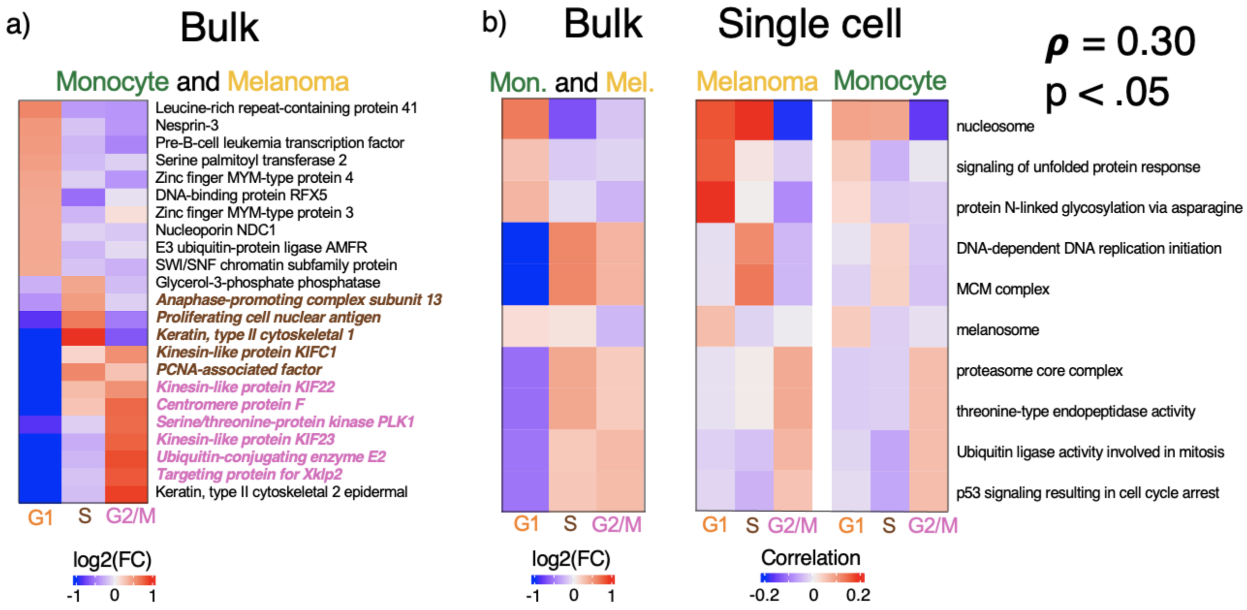

Figure S14 | Cell cycle analysis quality controls. (**a**) Proteins found to be differential from both monocyte and melanoma bulk CDC fractions. Proteins previously reported to be involved in S phase are colored in brown and previously associated G2 proteins are colored in pink. (**b**) Protein set enrichment analysis for bulk data based off protein fold changes between three CDC fractions and corresponding significant protein sets from correlation based single cell protein set enrichment analysis. The two analysis are significantly correlated suggesting agreement between bulk and single cell analysis.

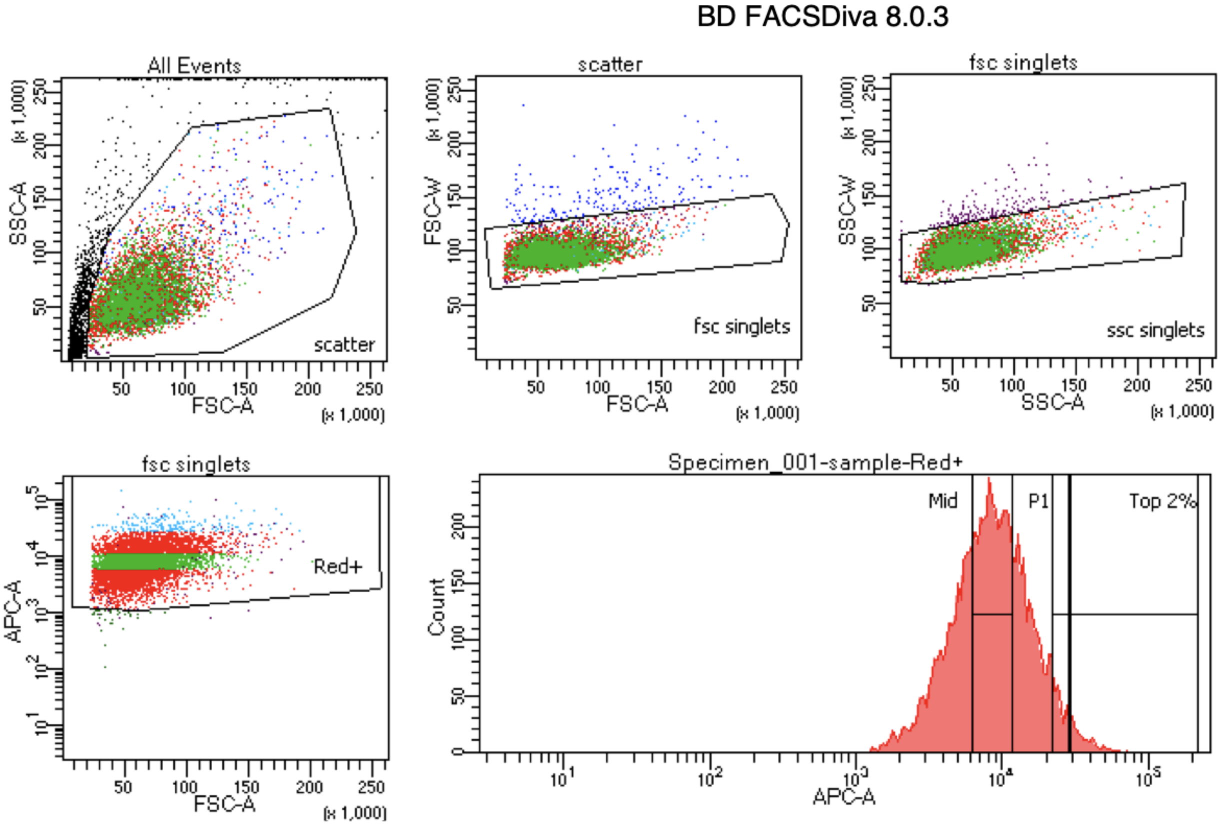

Figure S15 | FACS data used to sort melanoma subpopulation for glycogen assay. 2 populations were sorted based off red florescence intensity after appropriately gaiting for singlets.

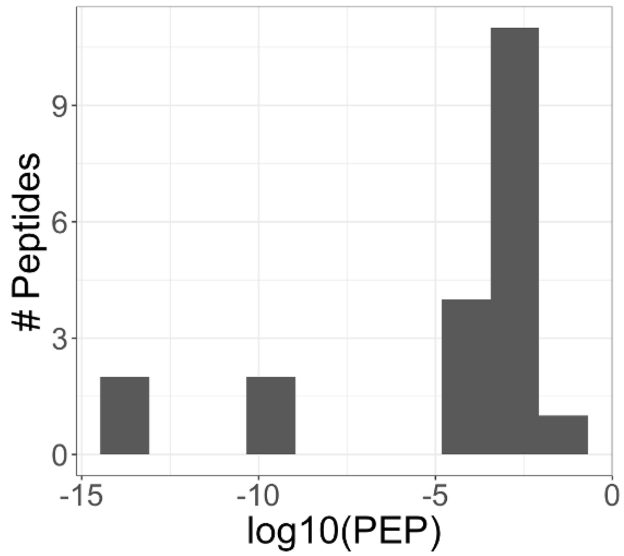

Figure S16 | A search with a fasta database containing all uniprot proteins identified 15 non-unique peptides that were not present in the over 80,000 non-unique peptides identified from the search with a fasta database that contained only the prioritized proteins. The posterior error probabilities (PEPs) for these 15 peptides are displayed as a histogram.

References

1. Huffman, G., Chen, A. T., Specht, H. & Slavov, N. DO-MS: Data-Driven Optimization of Mass Spectrometry Methods. *J. of Proteome Res.* **18,** 2493–2500 (6 2019).
2. Petelski, A. A. *et al.* Multiplexed single-cell proteomics using SCoPE2. *Nature Protocols* **16,** 5398–5425 (2021).
